# Prenatal diagnosis and genetic counseling of a case with trisomy 20 mosaicism and mixed-type maternal UPD20

**DOI:** 10.1016/j.plabm.2025.e00495

**Published:** 2025-08-02

**Authors:** Yun Huang, Fang Li, Xiaofeng Li, He Wang

**Affiliations:** a Department of Laboratory Medicine, Peking University Shenzhen Hospital, Shenzhen, China; b Guangdong Provincial Clinical Research Center for Laboratory Medicine, China

**Keywords:** Trisomy 20 mosaicism, Mixed-type maternal UPD20, Prenatal diagnosis, Multidisciplinary approach, Genetic counseling

## Abstract

**Background:**

To genetically analyze a prenatal specimen exhibiting mosaic 20q11.2 microdeletion syndrome with uniparental disomy of chromosome 20 (UPD20). The aim is to summarize the symptoms and prognosis of fetuses with this condition and provide guidance for genetic counseling and prenatal diagnosis in similar cases.

**Methods:**

Chromosomal karyotyping and Chromosomal Microarray Analysis (CMA) were performed on prenatal amniotic fluid specimens and peripheral blood samples from the parents.

**Results:**

The karyotype analysis of the fetal amniotic fluid cells revealed a mosaic pattern of 46,XN,+20[80]/46,XN[20], indicating an 80 % mosaicism ratio. The CMA results showed arr20p13q13.33(61,662–62,913,645)x2-3 mos with an 11 % mosaicism ratio and arr20p12.2q13.2(9,484,368–50,586,616)x2 hmz, inherited from the mother.

**Conclusion:**

Through interdisciplinary team discussion and analysis of a 42-year-old pregnant woman's fetus exhibiting mosaic 20q11.2 microdeletion syndrome with mixed-type maternal UPD20, relevant genetic counseling was provided to the pregnant woman, assisting in informed decision-making. The reporting of this case is significant for summarizing the symptoms and prognosis of fetuses with this condition and guiding prenatal diagnosis and genetic counseling in similar cases.

## 1 Introduction

Trisomy 20 syndrome (T20), particularly the complete form, is a rare and lethal chromosomal aberration. In contrast, the mosaic form of T20 is relatively common among prenatal diagnoses, occurring in approximately 1/7000 pregnancies and accounting for 16 % of all mosaic cases diagnosed prenatally [1,2]. Approximately 90 %–93 % of fetuses with mosaic T20 present with no abnormal phenotypes, while approximately 6.5 % exhibit associated phenotypes. Genetic counseling for mosaic T20 can be challenging, requiring a comprehensive analysis of various factors, including mosaic ratio, fetal ultrasound screening, and the presence of uniparental disomy (UPD) [3].

In this study, we present a case of prenatal diagnosis in a 42-year-old pregnant woman, who underwent chromosomal karyotype analysis and chromosomal microarray analysis (CMA) to investigate suspected trisomy 20 mosaicism and mixed-type maternal UPD20. The aim of this study was to provide evidence for genetic counseling and prenatal diagnosis in cases of trisomy 20 mosaicism and mixed-type UPD20.

## 2 Materials and methods

### 2.1 Ethical considerations

This study was conducted with the approval of the Ethics Committees of Peking University Shenzhen Hospital (Peking University Shenzhen Hospital Ethical Approval [Research] [2025] No. 188). We certify that the study was performed in accordance with the 2013 Declaration of Helsinki, upholding ethical standards in research involving human subjects. By addressing these ethical considerations, we reinforce the credibility of our study and the integrity of our findings.

#### 2.1.1 Subjects

The subject is a 42-year-old pregnant woman who has had two induced abortions in the past and is currently in her third pregnancy, which is natural. Her maternal grandmother had hypertension and diabetes, but there is no history of consanguineous marriage or significant adverse environmental exposures in her family. The woman's pregnancy has been uneventful so far, and she has a history of regular menstrual cycles. Due to her advanced maternal age, she underwent prenatal diagnosis at 19+ weeks of gestation, with amniocentesis performed under ultrasound guidance after obtaining informed consent.

The woman is generally in good health, with no personal history of hypertension, diabetes (prior to this pregnancy), heart disease, hepatitis, tuberculosis, syphilis, or other infectious diseases. However, she was diagnosed with gestational diabetes during her current pregnancy. There is no consanguineous relationship between the pregnant woman and her husband.

#### 2.1.2 Auxiliary examinations

**Fig. 1:**
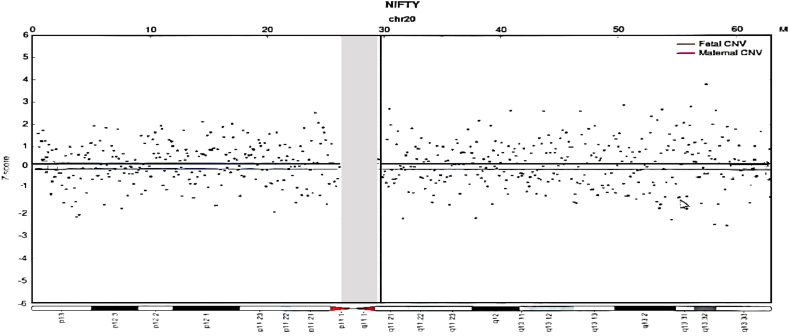
Non-invasive prenatal screening (NIPT) sequencing array for chromosome 20.

Ultrasound examination at **13 weeks of gestation** revealed a **nuchal translucency (NT) thickness of 1.6 mm** (reference range: <3.0 mm for singleton pregnancies), and no structural abnormalities were observed on the mid-pregnancy ultrasound. Prenatal serological screening at **16 + 4 weeks** reported a **T21 risk of 1/372**, exceeding the widely accepted critical risk threshold for Down syndrome (defined as risk <1/270).Non-invasive prenatal screening indicated a z-score of “+4.325” for chromosome 20, indicating a slight excess of this chromosome. However, the mosaic ratio did not reach 50 %, which did not meet the standard requirements for formal reporting, as shown in Fig. 1.

#### 2.1.3 Sample collection

After obtaining full informed consent, 20mL of amniotic fluid was collected for karyotype analysis, and an additional 10mL was utilized for chromosomal microarray analysis (CMA). The CMA results indicated the presence of partial homozygous regions on chromosome 20. Subsequent to this finding, and after obtaining further informed consent, 2mL of peripheral blood was collected from both parents for fetal CMA familial analysis. This study was approved by the Prenatal Diagnosis Technology Ethics Committee of our hospital.

#### 2.1.4 Chromosome Karyotype analysis

After centrifugation of the collected amniotic fluid, the sediment was collected, and 1–2mL of this sediment was inoculated into amniotic fluid cell culture medium. Karyotype analysis was performed after culturing, media changes, passages, harvesting, chromosome preparation, and G-banding. A minimum of 20 metaphases were counted by two observers, and at least 5 mid-phase karyotypes were analyzed. The diagnostic criteria for detecting mosaics followed the "Prenatal Genetic Diagnosis and Genetic Counseling of Chromosome Mosaics," and karyotype descriptions adhered to the "International System for Human Cytogenetic Nomenclature (ISCN2020)."

#### 2.1.5 Chromosomal microarray analysis

Ten milliliters of amniotic fluid samples with qualified quality control were collected aseptically. Genomic DNA was extracted using the QIAamp®DNA Blood Mini Kit (QIAGEN, Germany). The genomic DNA was digested into short fragments, adapters were added, and PCR amplification was performed. The products were purified using a magnetic bead method. The purified products were fragmented into 25-125bp fragments and biotinylated. The products were mixed with hybridization solution, denatured, and then hybridized to the chip, washed, and stained. The Affymetrix CytoScan 750K microarray platform (Thermo Scientifc, USA) was used to scan the chip for genome-wide chromosome copy number detection.

#### 2.1.6 Data analysis

All chromosomal microarray detection data were processed and analyzed using Chromosome Analysis Suite (ChAS) V4.3 software. This software can detect clinically relevant genes and copy number variations (CNVs) and loss of heterozygosity (LOH) with a genomic resolution greater than 100kb. UPD tool statistics [14] were used to analyze familial genotypes and generate images. The assessments of CNVs and LOH were referenced against the "Technical Standards and Guidelines for Interpretation and Reporting of Primary Copy Number Variants: Joint Consensus Recommendation of the American College of Medical Genetics and Genomics (ACMG) and Clinical Genome Resources (ClinGen)" from 2019 and the "Guidelines for the Application of Chromosomal Microarray Analysis Technology in Prenatal Diagnosis (2023)."

## 3 Results

### 3.1 G-banded Chromosome Karyotype of Amniotic Fluid Cells

**Fig. 2:**
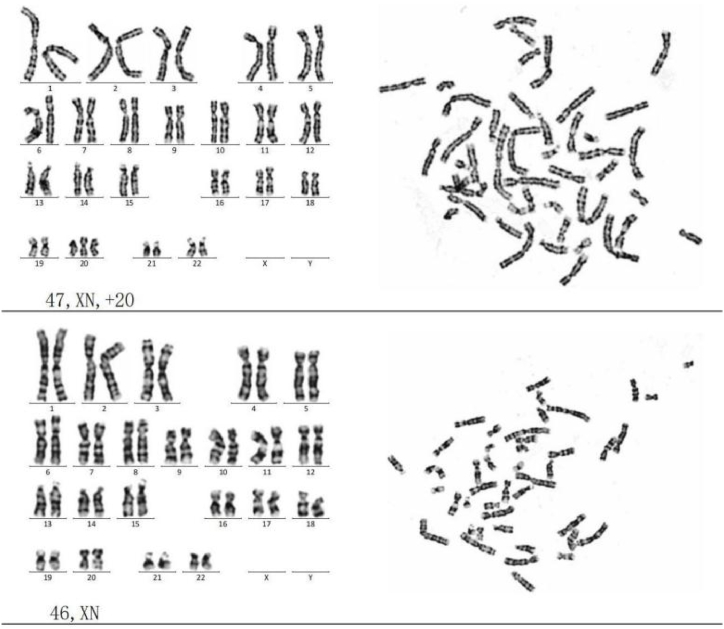
G-banded chromosome karyotype of amniotic fluid cells.

Counting of 100 metaphases and analysis of 10 karyotypes from **amniotic fluid cell** showed a karyotype of mos 47,XN,+20[80]/47,XN[20], with a mosaic proportion of 80 % (Fig. 2).

### 3.2 Chromosomal Microarray Analysis Results

**Fig. 3:**
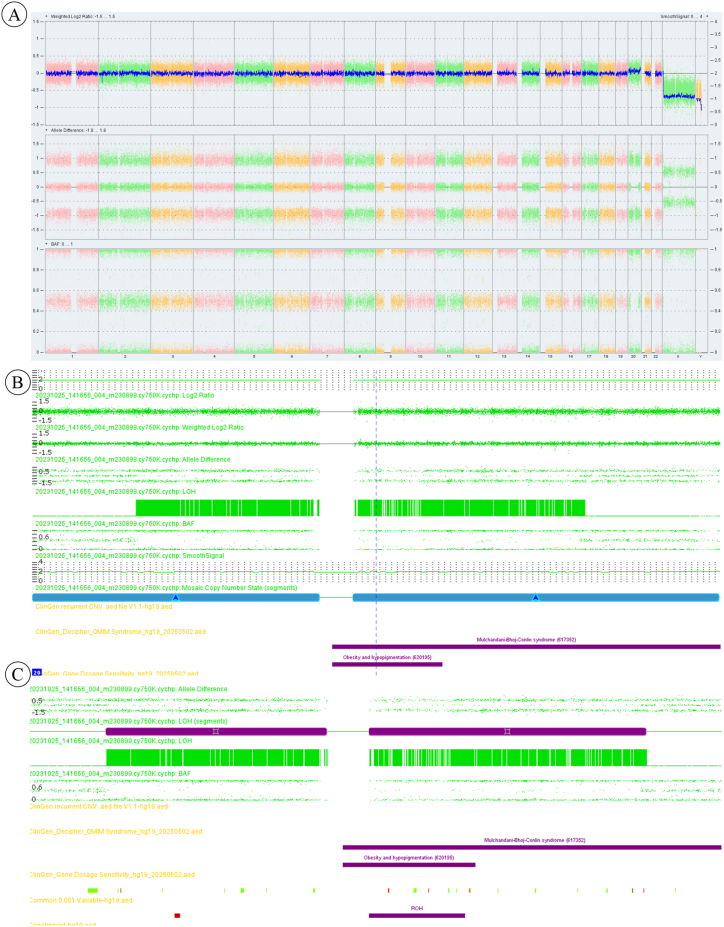
Chromosomal Microarray Analysis Results of Uncultured Amniotic Fluid Cells.Note:CMA detected approximately 11 % of fetal cells with trisomy of chromosome 20, and also showed UPD of the intermediate segment of chromosome 20 from one parent. Ⓐ:WGV diagram of the analysis result of chromosome microarray in amniotic fluid cells; Ⓑ:The CMA results of amniotic fluid samples indicated the detection of a 59.73Mb chimeric duplication region in the 20p13q13.33 section, (hg19) arr20p13q13.33 (61,662–62,913,645) × 2–3 mos(ratio = 2.11); Ⓒ:The CMA results of amniotic fluid samples indicated that 37.86Mb of heterozygous deletion (LOH) was detected in the 20p12.2q13.2 segment, (hg19) arr20p12.2q13.2(9,484,368–50,586,616) × 2 hmz.

Fetal CMA detected arr20p13q13.33(61,662–62,913,645)x2-3 mos, approximately 59.73Mb in size, with a mosaic proportion of 11 %; it also showed arr20p12.2q13.2 (9,484,368–50,586,616)x2 hmz, approximately 37.86Mb in size, as shown in Fig. 3. The presence of imprinted genes on chromosome 20 and the parental origin of the UPD segment may lead to phenotypic differences [4].

### 3.3 Mosaic proportion

As both karyotype analysis and chromosomal microarray results showed mosaic trisomy, the laboratory did not re-sample for amniotic fluid cell interphase FISH validation. Amniotic fluid cell karyotype analysis involves a significant impact of dominant growth during culture on mosaic proportion results, while CMA testing specimens are uncultured amniotic fluid cells. Therefore, the mosaic proportion is closer to the mosaic proportion of fetal.

### 3.4 Chromosomal Microarray Familial Analysis results

**Fig. 4:**
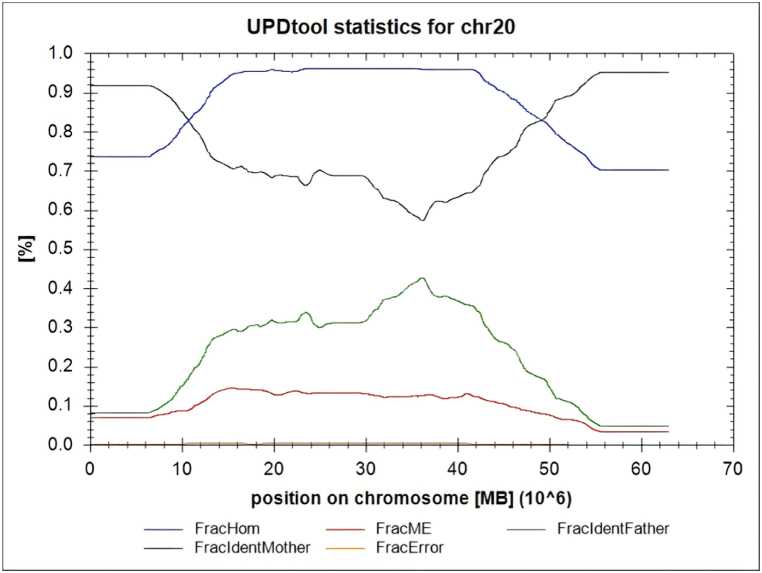
**Chromosomal Microarray Familial Analysis UPDtool Statistics**.Note: FracHom represents the proportion of homozygous regions in the fetus; FracldentMother represents the proportion of genotypes identical to the mother; FracldentFather represents the proportion of genotypes identical to the father; FracME represents the Mendelian Error Rate; FracError represents the genotyping error proportion.

Through the analysis of Single Nucleotide Polymorphism (SNP) loci in both parents and the fetus, it was determined that the fetus has a mixed-type maternal uniparental disomy (UPD) of chromosome 20, characterized by uniparental heterodisomy at both ends of the chromosome and uniparental isodisomy in the middle segment. However, due to the presence of a mosaicism ratio, there is a certain range in the proportion of the homozygous region in the middle segment of the chromosome that resembles the father's genetic pattern, which is more consistent with the actual situation, as illustrated in Fig. 4.

### 3.5 Pregnancy outcome

After multi-disciplinary consultation and genetic counseling, the potential risks were explained to the couple. Considering their age and other factors, they decided to continue the pregnancy. **Ultimately, she delivered a healthy male infant.**

## 4 Discussion

Trisomy 20 mosaicism is an extremely rare and lethal chromosomal aberration, with no reported cases of live births in the existing literature. However, some researchers believe that the trisomic cells detected in amniotic fluid may be limited to specific fetal tissues, leading to a relatively better prognosis [[2], [3], [4], [5], [6]]. There are also several reports in the literature that trisomy 20 mosaicism can result in a wide range of clinical manifestations and developmental abnormalities. The severity varies among individuals, with some fetuses showing no obvious phenotypic abnormalities, while others may have intellectual developmental delay, heart defects, neural tube defects, and other issues [[6], [7], [8], [9]].

Trisomy 20 mosaicism is associated with uniparental disomy (UPD) [1,11,12]. UPD20 can be either maternally or paternally derived, but maternally derived UPD20 is more common. UPD20 can lead to gene dosage imbalance on chromosome 20, further affecting fetal development. Paternally derived UPD (pUPD) can result in conditions such as neonatal hyperbilirubinemia and pseudohypoparathyroidism 1B (PHP1B). Maternally derived UPD (mUPD) can lead to Mulchandani-Bhoj-Conlin syndrome, manifesting as intrauterine growth restriction and abnormal postnatal growth and development [4]. In this study, prenatal diagnosis was performed on a fetus of a 42-year-old pregnant woman, revealing trisomy 20 mosaicism and maternally derived UPD20 genetic variation. Chromosome karyotype analysis and chromosomal microarray analysis were used to determine the genetic variation in the fetus. Karyotype analysis assesses the mosaic proportion by culturing amniotic fluid cells and observing the karyotype.

In this study, karyotype analysis showed a mosaic proportion of 80 % in the fetus. However, it is important to note that karyotype analysis involves cell culture, which can lead to uncertainties in the mosaic proportion due to the influence of dominant growth during culture. In contrast, chromosomal microarray analysis is a high-resolution technique based on DNA fragments that can detect copy number variations and regions of loss of heterozygosity (LOH) on chromosomes. In this study, CMA analysis showed a mosaic proportion of 11 % in the fetus, along with mixed-type UPD of chromosome 20 derived from the mother. Although the mosaic proportion from CMA was lower than that from karyotype analysis, the CMA results are more reliable and accurate as they are based on direct DNA analysis and are not affected by cell culture.

The Consensus Guidelines for Prenatal Diagnosis and Genetic Counseling of Chromosomal Chimerism in China [13] clearly recommend a prenatal chimerism detection strategy that combines karyotype analysis with CMA/CNV-seq, thereby ensuring simultaneous verification of chromosomal structural abnormalities and high-sensitivity detection of low-level chimerism. According to this standard, our research simultaneously employed the karyotypes of cultured amniotic cells and uncultured amniotic cells, successfully confirming fetal 20-chromosome chimeric trisomy and maternal 20-chromosome haploid (UPD20). It is worth noting that CMA detected a chimerism rate of 11 % in uncultured amniotic fluid cells, indicating that it can more accurately reflect the level of chimerism in vivo. This advantage stems from the direct genomic DNA analysis of CMA, which avoids the culture-induced clonal selection bias inherent in traditional cytogenetic methods. Therefore, the comprehensive application of karyotype and CMA can significantly improve the diagnostic accuracy and reliability of prenatal genetic assessment.

Compared to the studies by Jin Chunyan et al. [2] and Zhao Junhong et al. [6], our results exhibit some differences. The accuracy of our detection of trisomy 20 mosaicism varied from those reported in these studies. One possible reason is the differences in experimental details between our laboratory's CMA and karyotype analysis methods. Additionally, sample characteristics, such as the range of mosaic proportions or the presence of other genetic variations in different cases, may contribute to the observed differences. Technical limitations and variations in laboratory conditions may also affect the results. This underscores the importance of caution when applying research results to clinical practice, considering the differences and limitations among studies.

Multiple studies [1,[6], [7], [8], [9], [10], [11]] have documented the phenotypic manifestations of children with trisomy 20 chimera were recorded, revealing significant differences in the detection of this chromosomal aberration. As shown in Table 1, the proportion of mosaicism ranges widely among these cases, varying from less than 5 % to over 90 %. Furthermore, factors such as tissue origin and genetic testing methods may influence the results. The low proportion of trisomy 20 chimeras in amniocentesis is relatively favorable for pregnancy outcomes in the absence of UPD20 [1].

**Table 1:** Reported cases of children with 20q11.2 microdeletion syndrome.

reference documentation	age	sex	The karyotype of amniotic fluid cells	The source of tissue cells	clinical manifestation	UPD20
[8]	9 years old	girl	I: 83 %20 for trisomy II: 57 % trisomy 20	The villous tissue:15 % (4/26); Urinary cells: 25 % (3/12); Skin of buttocks:0 %(0/50); Umbilical cord blood:0 % (0/30); Kidneys, ureters, and bladder 0 %	Skin striped hypopigmentation; normal mental development	–
	8 years old	girl	90 % trisomy 20	Spinal cord:10 %(4/40); amniotic membrane:50 %(5/10); placenta:100 %(10/10); urine:50 %(50/50); cord blood:0 %; the left palm:30 % (6/20)	Recurrent eye infections; amblyopia, mild myopia; skin vortex pigmentation and hypopigmentation; normal intelligence; good exercise ability	–
[7]	Pregnant 38 weeks	boy	The villus:95 %	–	No abnormalities in growth and development	–
	induced labour	–	76 %	–	–	–
	Pregnant 39 weeks		4 %	–	Pregnant 38 weeks	–
[1]	Pregnant 38 weeks	boy	Week 16:15 % (3/20) Week 23:3.5 % (1/28)	cord blood:0 %(0/40)	Born healthy	excluded
[9]	11 years old	girl	>90 %	Refjection of tissue karyotyping	(Infant)motor retardation, intermittent esotropia; (2years old)language impairment; (4years old)constipation; recurrent urinary tract infection, skin pigmentation; (5years old)decreased flexibility: normal movement, normal cognition: (10 years old)outstanding ballet dancer, mild lower oblique fissure, posterior jaw,chest stenosis, shoulder tilt, hypotonia, skin pigmentation	–
	15years old	girl	not examined	skin flbroblast:90 %(18/20)	Low conical position, congenital bony cervical stenosis, thoracic and lumbar disc herniation, normal motor and cognitive development, learning disabilities, linear and wheeled nevus hypophyelanosis, constipation,myopia, hypotonia	–
	8years old	boy	>70 %	cord blood:0 %; placenta:50 %(10/20); skin flbroblast:35 %(7/20)	(At birth) mild facial asymmetry, oblique cephaly; (17 months)bradkinesia; (32 months) language delay, astigmatism: (Four and a half years old)leg mild asymmetry, poor coordination, dance; (Five and a half years old) hypopigmentation and pigmentation; (8 years old)social and emotional development delay, bilateral hip dysplasia, constipation,low blood pressure	–
[6]	induced labour	–	30 %	–	–	Yes
[10]	4 years old	boy	normal	The villus tissue before birth:trisomy 20 mosaicism	Wide anterior fontanelle, bilateral eyelid edema, unilateral cryptorchidism, feeding difficulties	Yes(Maternal)
	5 years old	boy	–	–	(infancy) bilateral cryptorchidism, bilateral temporal stenosis, feeding difficulties, hypotonia, (from 19 months) developmental delay	Yes(Maternal)
[11]	Born	–	98 %	Placenta and urine deposits:100 %; Peripheral blood: (72h) 0 %; (24h) 10 %	(Pregnant 36 weeks) intrauterine growth retardation, oligohydramnios; (neonatal period) mild systolic heart murmur; (9 months) psychomotor retardation, mild facial deformity	Yes(Maternal)

Ongoing follow-up of these patients is crucial, as clinical manifestations and development may vary over time. Close monitoring of growth and development, cardiac function, and neurological development is essential. Regular physical examinations, developmental assessments, echocardiograms, and neuroimaging studies allow for the early detection and management of any concerns. Comprehensive care and support, optimized through follow-up, aim to improve the overall management, treatment, and quality of life for these patients.

In summary, genetic analysis of this case, coupled with interdisciplinary team discussion, has provided genetic counseling and decision support for the pregnant mother. Additionally, we have summarized the symptoms and prognosis of mosaic T20 with maternal UPD20, offering guidance for prenatal diagnosis and genetic counseling in similar cases. However, it is important to note that this study involves only one case and a newborn fetus, necessitating further research, clinical data support, and longitudinal follow-up. Such efforts will facilitate a deeper understanding of the characteristics, treatment modalities, and long-term outcomes of this rare condition. Encouraging interdisciplinary collaboration and communication is crucial to advancing our comprehensive understanding and effective management of these complex disorders.

## CRediT authorship contribution statement

**Yun Huang:** Writing – review & editing, Writing – original draft, Visualization, Validation, Supervision, Software, Resources, Methodology, Investigation. **Fang Li:** Data curation. **Xiaofeng Li:** Project administration, Funding acquisition. **He Wang:** Formal analysis, Data curation.

## Declaration of competing interest

We declare that there are no conflicts of interest inrelation to the manuscript titled "[Prenatal Diagnosis and genetic counseling of a Case with Trisomy 20 Mosaicism and mixed-type maternal UPD20]" submitted to[Practical Laboratory Medicine]. I confirm that the results and interpretationsreported in the manuscript are original and have not beenplagiarized.

## Data Availability

Data will be made available on request.
